# Draft Genome Sequence of *Agreia bicolorata* Strain AC-1804, a Producer of Large Amounts of Carotenoid Pigments, Isolated from Narrow Reed Grass Infected by the Phytoparasitic Nematode

**DOI:** 10.1128/genomeA.01383-15

**Published:** 2015-12-03

**Authors:** Ilya Vasilyev, Maria Siniagina, Sergey Malanin, Eugenia Boulygina, Tatiana Grygoryeva, Dina Yarullina, Olga Ilinskaya

**Affiliations:** Institute of Fundamental Medicine and Biology, Kazan (Volga Region) Federal University, Kazan, Russia

## Abstract

Here, we report the draft genome sequence of *Agreia bicolorata* strain AC-1804, isolated from narrow reed grass galls induced by a plant-parasitic nematode which is able to produce large amounts of carotenoid pigments. The draft genome sequence of 3,919,485 bp provides a resource for carotenoid pathway research.

## GENOME ANNOUNCEMENT

Coryneform bacteria (or diphteroids) belong to a group of large, ecologically diverse, and partially studied nonspore-forming polymorphic Gram-positive rod-shaped actinobacteria (1). The first strain of *Agreia bicolorata* AC-1804, a new taxon of coryneform bacteria, was described in 2001 (2). The strain source, narrow reed grass (*Calamagrostis neglecta*) galls infected by the nematode *Heteroanguina graminophila*, were collected in the Moscow Region in June 1991. The *Agreia* genera contain 2 species and about 60 undefined strains. 16S rRNA sequences of the *Agreia* genera have been found on ice wedges of Alaskan glaciers (3), on flower petals in Germany (4), inside crabs from a west Korean bay (5), in rye doughs (6), etc. The main feature of the AC-1804 strain is the production of large quantities of yellow and red pigments, while most of the other coryneform bacteria produce yellow pigment only.

The sequencing of *A. bicolorata* AC-1804 was performed on a 454 Life Sciences GS Junior system (Roche Applied Science, USA), with approximately 24-fold genome coverage. The obtained 143,732 reads were assembled with two *de novo* genome assemblers: the proprietary GS De Novo Assembler (Newbler software suite), v3.0 and the free SPAdes assembler, v3.1.1. The assembly statistics include 143,085 (99.55%) survived reads, 25 contigs with 348,834 bp *N*_50_ size, and total length 3,912,036 bp for the Newbler assembly, and 143,688 (99.97%) survived reads, 21 contigs with 708,433 bp *N*_50_ size, and total length 3,919,485 bp for the SPAdes assembly. In both cases, G+C content was 65.2 mol% and all contigs contained no plasmids. Therefore, the SPAdes assembly was annotated on two pipelines: the classic RAST (Rapid Annotation using Subsystem Technology) server and the best-placed reference protein set GeneMarkS+ (NCBI Prokaryotic Genome Annotation Pipeline) v2.9 (revision 460460). The annotation consists of 3,712 predicted CDSs and 51 RNAs for the RAST draft, and 3,111 CDSs, 46 tRNAs, 5 rRNAs, and 1 noncoding RNA (ncRNA) for the NCBI draft. Both drafts are complementary. The number of unknown proteins is large, up to 25% of all CDSs.

The obtained data helped to discover a gene cluster of rare C50 carotenoid biosynthesis. Genome and genome comparison analysis with other fully and partially sequenced coryneform bacteria demonstrated spread and diversity of the found gene cluster, especially in psychrophilic organisms (data not shown).

### Nucleotide sequence accession number.

SPAdes shotgun genome assembly has been deposited in GenBank under the accession number JYFC00000000.
